# The impact of nirsevimab prophylaxis on RSV hospitalizations: a real-world cost-benefit analysis in Tuscany, Italy

**DOI:** 10.3389/fpubh.2025.1604331

**Published:** 2025-07-03

**Authors:** Vieri Lastrucci, Martina Pacifici, Giorgia Alderotti, Monia Puglia, Elettra Berti, Federica Barbati, Lorenzo Lodi, Silvia Boscia, Francesco Nieddu, Giuseppe Indolfi, Diego Peroni, Marco Martini, Chiara Azzari, Fabio Voller, Maria Moriondo, Silvia Ricci

**Affiliations:** ^1^Epidemiology Unit, Meyer Children’s Hospital IRCCS, Florence, Italy; ^2^Epidemiologic Observatory, Regional Healthcare Agency of Tuscany, Florence, Italy; ^3^Neonatal Intensive Care Unit, Meyer Children’s Hospital IRCCS, Florence, Italy; ^4^Pediatrics and Neonatology Unit, Santo Stefano Hospital, USL Toscana Centro, Prato, Italy; ^5^Department of Neurofarba, University of Florence, Florence, Italy; ^6^Immunology Unit, Meyer Children’s Hospital IRCCS, Florence, Italy; ^7^Pediatric and Liver Unit, Meyer Children’s Hospital IRCCS, Florence, Italy; ^8^Pediatric Clinic, Department of Clinical and Experimental Medicine, University of Pisa, Pisa, Italy; ^9^Pediatric Unit, San Donato Hospital, Arezzo, Italy; ^10^Department of Health Sciences, University of Florence, Florence, Italy

**Keywords:** respiratory syncytial virus (RSV), immunization, prevention, nirsevimab, hospitalizations, cost–benefit analysis

## Abstract

**Background:**

Respiratory Syncytial Virus (RSV) is the leading cause of hospitalizations in infants. The approval of nirsevimab, a long-acting monoclonal antibody, has extended the potential for RSV prophylaxis to all infants. This study assesses the cost–benefit of various Nirsevimab prophylaxis strategies for infants during their first RSV season in preventing RSV-associated hospitalization in the Tuscany region, Italy.

**Methods:**

The analysis was conducted from the perspective of the healthcare payor. Real-world data from the Tuscany birth cohort (*N* = 21,017) experiencing their first RSV season in the 2023/2024 season were used to calculate the net benefit and benefit cost ratio (BCR) of three possible nirsevimab prophylaxis strategies compared with prophylaxis practices at the time of the study, which includes the use of palivizumab in eligible infants. RSV-associated hospitalizations and severe hospitalizations were considered as health outcomes. Sensitivity analyses were performed to identify influential variables.

**Results:**

Under prophylaxis practices at the time of the study, there were a total of 663 hospitalizations associated with RSV, including 102 severe cases, representing €5,247,645 in costs. An extended prophylaxis strategy with nirsevimab, including all infants born both before and during the RSV season, resulted in the highest number of hospitalizations avoided (378), with a BCR close to break-even (0.96). A seasonal-only strategy targeting infants born during the season prevented the fewest hospitalizations (252), showing a positive BCR of 1.15. Finally, a seasonal strategy with targeted catch-up, including also preterm infants born before the season, yielded the highest cost–benefit ratio (1.56), preventing 270 hospitalizations.

**Conclusion:**

Universal prophylaxis strategies with nirsevimab, targeting all infants during their first RSV epidemic season, substantially reduce hospitalization burdens without increasing economic pressure on the healthcare system. Although alternative strategies are more cost-effective, they prevent fewer hospitalizations, emphasizing the public health value of broader prophylaxis approaches.

## 1 Introduction

Respiratory syncytial virus (RSV) is the major cause of a broad spectrum of respiratory infections in children worldwide, ranging from mild upper respiratory tract infections to severe conditions like bronchiolitis and pneumonia (1). RSV represents the primary cause of hospitalizations due to respiratory infection in infants, with the highest incidence in the first months of life, leading to an important disease burden and mortality (2). Moreover, early-life RSV infection may result in long-term comorbidities such as recurrent wheezing, decreased lung function and asthma (3–5).

The seasonal trend of RSV is variable in different climatic setting; in temperate regions RSV infections primarily occur from the beginning of October to the end of March.

Although most infections occur in otherwise healthy infants, underlying comorbidities such as prematurity and severe cardiac or pulmonary disease significantly increase the risk of contracting severe RSV infections (6, 7).

During the epidemic season, RSV-related hospitalizations place a significant burden on pediatric healthcare facilities. This phenomenon was particularly evident and severe following the end of containment measures introduced during the Severe acute respiratory syndrome coronavirus 2 (SARS-CoV-2) pandemic, so that a notable resurgence in annual RSV infections and subsequent hospital admissions was demonstrated in post-pandemic years (8).

Until 2023, the only available preventive strategy for RSV infection relied on the use of palivizumab, a recombinant humanized monoclonal antibody that has demonstrated efficacy in preventing severe lower respiratory tract infections caused by RSV in at-risk neonates and infants. However, due to the high costs, palivizumab is only indicated for a restricted pediatric population, particularly pre-term infants and children with comorbidities such as hemodynamically significant congenital heart disease or bronchopulmonary dysplasia (9). Moreover, palivizumab requires repeated administrations -five monthly intramuscular injections- throughout the epidemic season, due to its short half-life.

Since the majority of RSV hospitalizations occur in healthy term-born children, the current prevention program has left most of the pediatric population (especially those under 2 years old, and particularly under 6 months old) vulnerable to developing severe illness.

To overcome these limitations, other strategies for RSV infection prevention have been identified including passive immunization (long-acting monoclonal antibodies and maternal vaccination during pregnancy) and active immunization (RSV vaccination in pediatric population).

Long-acting monoclonal antibodies can protect with a single administration for the entire epidemic season and exhibit high direct neutralizing activity against the F protein with higher affinity for RSV compared to palivizumab (10–12). These advantages would allow their use in all infants—regardless of their gestational age or underlying conditions. This would enable the implementation of universal prevention strategies targeting infants born during and before the epidemic season.

Currently, nirsevimab is the only licensed long-acting monoclonal antibody, that has demonstrated its safety and efficacy in preventing RSV infections in both healthy and preterm children. Randomized controlled trials have shown a significant reduction in RSV respiratory infections requiring medical assistance, in those requiring hospitalization, in very severe RSV infections but also in lower respiratory tract infections of all causes (13–16).

In the last epidemic season, some European countries (Spain, France, Luxemburg, and the Italian region Valle D’Aosta) and the United States have implemented the universal immunization with nirsevimab, demonstrating an excellent population coverage and an effectiveness ranging from 70 to 90% (17–23).

The Italian Life Calendar Vaccination Board as well as the Italian Societies of Pediatrics and Neonatology have expressed their support for the potential use of nirsevimab for universal prevention of RSV infections in newborns. However, no Italian regions, except for Valle D’Aosta, implemented it during 2023/2024 season (24).

Using data from an area-based cohort of infants from the Tuscany region (Italy) who would have been eligible for nirsevimab in the 2023/2024 epidemic season, the aim of the present study is to assess the cost and the specific benefit of implementing a prophylaxis programme with nirsevimab against the prophylaxis practices at the time of the study. The analysis examines all infants in the Tuscany region during their first RSV season, considering both RSV hospitalizations prevented and cost savings from the discontinuation of palivizumab.

## 2 Materials and methods

### 2.1 Overview

This cost–benefit analysis considered the implementation of an RSV prophylaxis programme with nirsevimab in the Tuscany region from the perspective of the healthcare payor, i.e., the Regional Health Care System. Specifically, three possible nirsevimab immunization strategies were compared against the prophylaxis practices in place at the time of the study in the Tuscany region, which includes the use of palivizumab only in eligible infants. The impact of the prophylaxis programs was measured using hospitalizations and severe hospitalizations for RSV-associated acute lower respiratory infections (ALRI) as health outcomes. The benefits of the interventions included the cost savings from avoided hospitalizations and discontinuation of palivizumab prophylaxis. The cost and the benefits of the intervention were assessed only for the first epidemic season, so that hospitalizations occurred in the following seasons due to recurrent wheezing or asthma were not evaluated. Real-world data from the Tuscany birth cohort during the 2023/2024 epidemic season were used both to calculate the health and cost outcomes of the scenario at the time of the study and to serve as the basis for calculating these outcomes in the nirsevimab immunization scenarios. The study was approved by the Tuscany Region Ethics Committee (No. 183–2020). No informed consent was required as anonymized administrative data were used.

### 2.2 Scenario at the time of the study

The scenario at the time of the study consists of up to five monthly administrations of palivizumab during the epidemic season for eligible infants (gestational age at birth <32 weeks, and infants with congenital or chronic diseases, i.e., bronchopulmonary dysplasia, chronic lung disease, haemodynamically significant congenital heart disease and immunodeficiency disorders), and no prophylaxis for other infants.

#### 2.2.1 Target population and data sources

According to the seasonality of RSV in Italy, the RSV epidemic season was defined as 1 October to 30 April. All infants aged 6 months and under who were resident in Tuscany before the start of the 2023/2024 epidemic season (April to September) and those born in Tuscany during the epidemic season (October to March) were identified from the regional Population Registry. This registry covers all residents of Tuscany and records departing and new residents, births and deaths. Infants born in April of the 2023/2024 epidemic season were not considered, given the low circulation of RSV, which does not support the use of prophylaxis in their first epidemic season.

These infants were followed up retrospectively to identify any hospitalizations due to lower respiratory tract infections associated with RSV through record linkage with the Hospital Discharge Registry using an anonymous unique identifier. Infants with missing data on gestational age at birth, those with missing or erroneous unique identifiers, and those who died or migrated out of Tuscany within the first week of life or before the start of the epidemic season were excluded from the analysis. Infants were followed-up until the end of the season (30th of April), death, or migration out of the Tuscany Region, whichever occurred first.

#### 2.2.2 Health outcomes and costs

Hospitalizations for RSV-associated ALRI were identified using the following ICD-IX CM specific diagnosis codes: 466.11, 480.1, and 079.6 code associated with any other ALRI codes (ICD-IX CM Codes: 466.0–466.19; 480–491.9; 518.81–518.84; 769; 770.84). A severe RSV hospitalization was defined as an in-hospital stay which required an intensive care unit admission or the use of mechanical ventilation.

Hospitalization-related costs and costs associated with palivizumab prophylaxis were considered. Specifically, the overall cost associated with RSV-related hospitalizations was calculated using the average cost of an RSV-associated hospitalization and a severe hospitalization in infants under 2 years of age, as retrieved from the Italian Health Network for Standard Costs, Indicators, and Results, which provides data on standard costs from a network of Italian hospitals. Only costs directly related to patient care were included in the calculation of the average hospitalization costs. The costs of administering palivizumab prophylaxis to the study population were obtained from the Tuscany Regional Registry of Drugs and Medical Devices.

### 2.3 Nirsevimab immunization scenarios

Three nirsevimab immunization scenarios were evaluated: seasonal with extended catch-up, seasonal with targeted catch-up, and seasonal-only prophylaxis programs. In the seasonal with extended catch-up program, all infants born during the epidemic season receive a dose of nirsevimab at birth, and infants born up to 6 months before the season are recalled to receive a dose at the start of the epidemic season. In the seasonal with targeted catch-up program, all infants born during the epidemic season receive a dose of nirsevimab at birth, and only moderate- and high-risk infants born up to 6 months before the season are recalled to receive a dose at the start of the epidemic season. Prematurity is linked to a higher likelihood of RSV-related complications. Infants were stratified according to their gestational age (GA) at birth as follows: high-risk infants (very preterm: <32 weeks of GA), moderate-risk infants (moderate to late preterm: 32–36 weeks of GA), and low-risk infants (term: ≥37 weeks of GA). Lastly, the seasonal-only scenario assumes that the prophylaxis program only covers all the infants born during the epidemic season. In all the scenarios nirsevimab was considered as a substitute for palivizumab in infants eligible to receive palivizumab.

#### 2.3.1 Assumptions and data inputs

Adherence to the prophylaxis program was assumed to be 71% for all infants (25), except for high-risk infants, for whom adherence was assumed to be 80% (25) (Table 1). This assumption was made to reflect real-world variations in uptake across risk groups, as prophylaxis adherence is expected to vary according to infants’ level of susceptibility and the perceived benefit of protection.

**Table 1:** Input parameters considered in the analysis.

Input	Value	Reference
Cost of a RSV-associated hospitalization (standard ward)	€ 4,296	Italian Health Network for Standard Costs, Indicators, and Results
Cost of RSV-associated severe hospitalization	€ 14,583	Italian Health Network for Standard Costs, Indicators, and Results
Cost of nirsevimab (unit cost per dose)	€ 230	Tuscany Region, 2024 (26)
Administration-related cost per dose in catch-up programs	€ 15	Tuscany Region, 2015 (27)
Wastage rate	5%	WHO, 2019 (28)
Nirsevimab adherence rate in high-risk infants	80%	Kieffer et al., 2022 (25)
Nirsevimab adherence rate in moderate- and low-risk infants	71%	Kieffer et al., 2022 (25)
Efficacy of nirsevimab on RSV-associated hospitalizations	82%	Ares-Gómez et al., 2024 (17)
Efficacy of nirsevimab on RSV-associated severe hospitalizations	86.9%	Ares-Gómez et al., 2024 (17)

Nirsevimab efficacy data reported in the population-based longitudinal study by Ares-Gómez et al. (17) were used, which estimated an 82% reduction in the prevention of RSV-associated hospitalizations and an 86.9% reduction in severe RSV-associated hospitalizations (Table 1).

The total costs associated with the administration of nirsevimab were calculated taking into account a single administration per child at a price of €230 per dose (26) (Table 1). For children born outside of the epidemic season, it was assumed that nirsevimab would be administered by family paediatricians, in the same way that routine childhood vaccinations are currently administered in Italy. The administration-related cost of prophylaxis was assumed to be the same as that currently reimbursed to family paediatricians for the administration of routine vaccinations in the Tuscany region (i.e., €15) (27). Prophylaxis for infants born during the epidemic season was assumed to be administered during hospitalization, and therefore no additional administration-related costs were considered in this case. Costs of drug wastage were calculated assuming a 5% wastage rate, as indicated by the World Health Organization (WHO) (28).

#### 2.3.2 Health outcome and cost estimation

Real-world data from the scenario at the time of the study were used to estimate hospitalizations and costs incurred under the nirsevimab immunization scenarios, along with assumptions about nirsevimab prophylaxis adherence and efficacy. Specifically, the adherence and efficacy rates were applied to the hospitalization rates of the scenario at the time of the study to estimate the number of RSV-associated hospitalizations and severe hospitalization that could be prevented in the nirsevimab immunization scenarios. Costs associated with RSV-associated hospitalizations were calculated as in the scenario at the time of the study. The total costs associated with the administration of nirsevimab were calculated taking into account adherence rates in different population groups.

### 2.4 Statistical analysis

Categorical variables were presented as frequencies and percentages, while continuous variables were described as median and interquartile range (IQR).

The seasonal cumulative number of RSV-associated hospitalizations was considered in each scenario. The seasonal cumulative number of hospitalizations was also adjusted to account for the fact that the hospital discharge registry of Tuscany does not include data on laboratory-confirmed RSV infections, which may lead to an underestimation of the true burden of RSV-related hospitalizations. To address this potential underreporting, both hospitalization and laboratory data from Meyer Children’s Hospital (MCH), a tertiary care hospital in the region, were used. Specifically, the proportion of laboratory-confirmed RSV in ALRI diagnoses without RSV coding was obtained from the MCH discharge and laboratory databases. This proportion – referred to as RSV underreporting adjustment factor - was then applied to all the hospitalizations with an ALRI diagnosis without RSV coding experienced by the study cohort in the Tuscany region, in order to estimate the potential number of RSV-related hospitalizations that were not coded as such. The adjusted number of RSV-associated hospitalizations was determined by adding these estimated cases to those already identified with RSV-specific ICD-9-CM codes, as previously described.

For the intervention scenarios, the costs of the prophylaxis program were aggregated by including the costs of drug use, the administration-related costs and drug wastage. The benefits of the nirsevimab prophylaxis programs included the monetary value of averted RSV-associated hospitalizations (non-severe and severe) and the savings associated with discontinuing the palivizumab prophylaxis program.

The net benefit - calculated as the subtraction of total cost from total benefit - and the benefit–cost ratio (BCR) - calculated as the ratio of the total benefit to the total cost - were estimated for all the nirsevimab immunization scenarios.

#### 2.4.1 Sensitivity analyses

Deterministic sensitivity analyses were performed to account for uncertainty in the assumptions made. Specifically, the following variables were varied within a plausibility range of +/− 20%: adherence rates, nirsevimab efficacy, hospitalization cost, severe hospitalization cost, price per dose of nirsevimab, nirsevimab administration-related cost, and RSV underreporting adjustment factor.

## 3 Results

A total of 21,471 infants were born before or during the 2023/2024 RSV epidemic season in Tuscany. Of these, 422 (1.96%) had a missing or erroneous unique identifier or missing data on gestational age at birth and 32 (0.15%) died or migrated out of the Tuscany region within the first week of life or before the start of the epidemic season and were excluded from the analysis. A total of 21,017 of infants were included in the study, of whom 10,479 (49.9%) were born during the epidemic season and 10,538 (51.1%) born before the epidemic season (Table 2).

**Table 2:** Characteristics of the study population (full cohort and in-season birth cohort).

	Full cohort *N* (%)	In-season birth cohort *N* (%)
Total	21,017	10,479
Male	10,780 (51.2)	5,363 (51.2)
Gestational age at birth		
<32 weeks	162 (0.8)	80 (0.8)
32–33 weeks	195 (0.9)	92 (0.9)
34–36 weeks	1,098 (5.2)	531 (5.1)
≥ 37 weeks	19,562 (93.1)	9,776 (93.3)

### 3.1 RSV hospitalization and economic burden in the scenario at the time of the study

During the epidemic season, a total of 569 RSV-associated hospitalizations were observed in the entire study population, of which 66 (11.6%) were severe hospitalizations (Table 3). Data on gestational age at birth, age at admission and length of hospital stay for the hospitalizations registered in the study cohort are shown in Supplementary Table 1. After adjustement for RSV undereporting, the total number of RSV-associated hospitalizations was 663, of which 102 (15.4%) were severe hospitalizations (Table 3). The cohort of infants born during the epidemic season recorded a total of 439 RSV-associated hospitalizations after adjustement for RSV undereporting.

**Table 3:** RSV-associated hospitalizations in the study population (unadjusted and adjusted for RSV underreporting).

	RSV-associated hospitalization (unadjusted)	RSV-associated hospitalization (adjusted)
Full cohort		
Total	569	663
Non-severe hospitalization	503 (88.4)	561 (84.6)
Severe hospitalization	66 (11.6)	102 (15.4)
In-season birth cohort		
Total	369	439
Non-severe hospitalization	314 (85.0)	351 (80.0)
Severe hospitalization	55 (14.9)	88 (20.0)

Overall, the total costs registered in the scenario at the time of the study, considering the adjusted total number of RSV-associated hospitalizations, amounted to €5,247,645, of which €3,897,522 was for in-hospital treatments and €1,350,123 for palivizumab prophylaxis.

### 3.2 Cost and impact of nirsevimab immunization programs on RSV hospitalization and economic burden

The seasonal with extended catch-up prophylaxis program was estimated to avert 289 non-severe RSV-associated hospitalizations and 40 severe hospitalizations during the epidemic season (Table 4). Taking into account the underreporting of RSV, the total number of RSV-associated hospitalizations averted was 378, of which 57 were severe hospitalizations (Table 4). Regarding the cost of the extended program, the estimated total cost of implementing the prophylaxis was €3,719,541, including €112,340 (3.02%) in administration-related costs (Table 4). The total cost of delivering this prophylaxis program exceeded the total benefits related to hospitalization prevention and palivizumab discontinuation, resulting in an estimated net benefit of -€541,637, or -€152,143 when accounting for underreporting of RSV. Considering underreporting of RSV, the estimated BCR was 0.96 - indicating that for every €1 spent on prophylaxis, approximately €0.96 is saved in hospital treatment and palivizumab costs (Table 4).

**Table 4:** Hospitalizations averted, total costs, total benefits associated with the nirsevimab prophylaxis programs (seasonal with extended catch-up, seasonal with targeted catch-up, and seasonal-only programs).

	Hospitalizations averted (severe hospitalizations)	Total costs of the prophylaxis program	Total benefits generated from hospitalization	Net benefit	Benefit - cost ratio
Seasonal with extended catch-up					
Unadjusted for RSV underreporiting	289 (40)	3,719,541 €	3,177,904 €	−541,637 €	0.85
Adjusted for RSV underreporiting	321 (57)	3,719,541 €	3,567,398 €	−152,143 €	0.96
Seasonal with targeted catch-up					
Unadjusted for RSV underreporiting	196 (35)	1,937,364 €	2,694,244 €	756,880 €	1.39
Adjusted for RSV underreporiting	219 (51)	1,937,364 €	3,028,226 €	1.090,862 €	1.56
Seasonal-only					
Unadjusted for RSV underreporiting	182 (33)	1,798,521 €	1,738,721 €	−59,799 €	0.97
Adjusted for RSV underreporiting	203 (49)	1,798,521 €	2,062,699 €	264,178 €	1.15

The seasonal with targeted catch-up prophylaxis program was estimated to avert 219 non-severe RSV-associated hospitalizations and 51 severe hospitalizations, taking into account the underreporting of RSV (Table 4). The estimated total cost of implementing prophylaxis was €1.937.364. Considering underreporting of RSV, the estimated net benefit of the program was € 1,090,862, and the estimated BCR was 1.56 - indicating that for every €1 spent on prophylaxis with nirsevimab, approximately €1.56 is saved in hospital treatment and palivizumab costs.

The seasonal-only prophylaxis program was estimated to avert 203 non-severe RSV-associated hospitalizations and 49 severe hospitalizations, after adjusting for underreporting of RSV (Table 4). The estimated total cost of implementing the seasonal-only prophylaxis program was €1,798,521. Considering underreporting of RSV, the estimated net benefit of the seasonal-only prophylaxis program was € 264,178, and the estimated BCR was 1.15.

### 3.3 Sensitivity analysis

**Figure 1:**
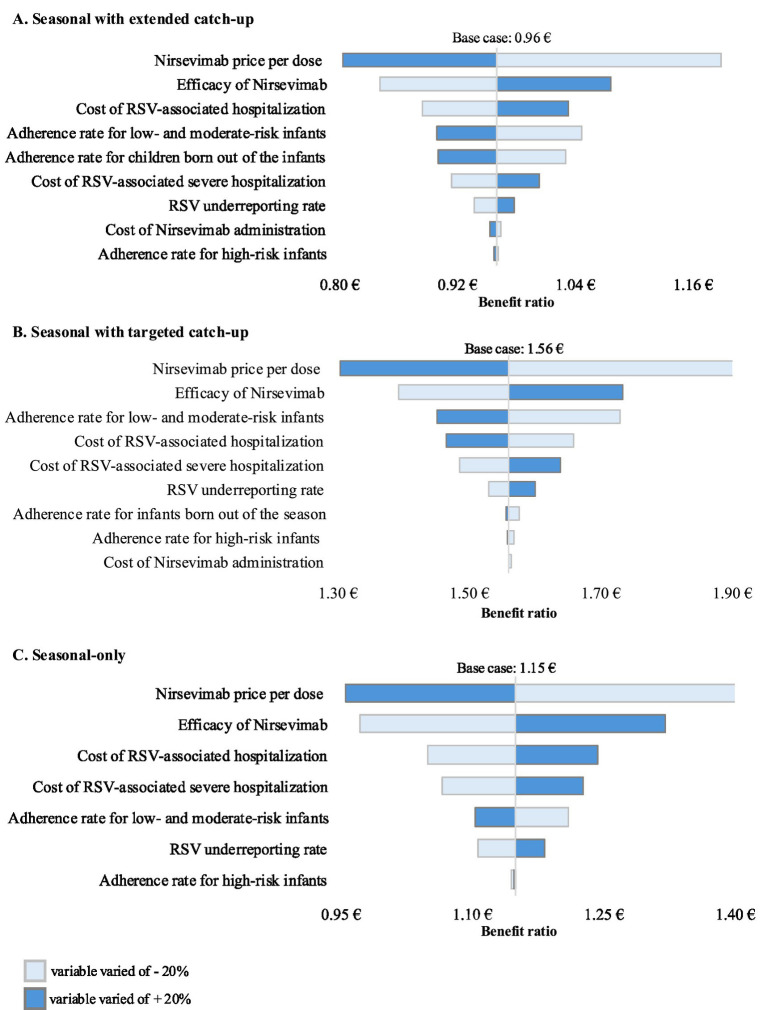
One-way sensitivity analyses of key variables influencing the cost–benefit ratio of Nirsevimab prophylaxis programs versus palivizumab prophylaxis practice. **(A)** Seasonal prophylaxis with extended catch-up program (base case benefit–cost ratio of 0.96), **(B)** Seasonal prophylaxis with targeted catch-up program (base case benefit–cost ratio of 1.56), and **(C)** Seasonal-only program (base case benefit–cost ratio of 1.15). Baseline parameters were varied by ±20% to reflect a reasonable estimate of their potential variability range.

Figure 1 shows the results of the sensitivity analyses of the variation in BCR for the three prophylaxis programmes considered when the baseline parameters were changed by +/− 20%. For all prophylaxis programmes, the parameters with the largest impact on the BCR, either positive or negative, compared to the base case were the price per dose of nirsevimab and the efficacy rate of nirsevimab. The seasonal with extended catch-up and the seasonal with targeted catch-up programs were also sensitive to changes in the level of adherence to prophylaxis in low-risk infants, whereas this parameter was less influential in the seasonal-only programme. Adherence to prophylaxis in out-of-season infants was also a relevant factor to consider in the seasonal with extended catch-up strategy.

## 4 Discussion

The urgency of implementing a new RSV preventive strategy has increased significantly following the resurgence in RSV-associated hospital admissions after SARS-CoV-2 pandemic, which has placed a consequent high disease burden on healthcare systems. This study provides the first real-world cost-benefit estimate conducted in the Tuscany region prior to the introduction of nirsevimab. It provides a potential assessment of the implications of universal prophylaxis with nirsevimab in terms of eligible population, the cost of the monoclonal antibody for healthcare systems, and its impact on preventing RSV-related hospitalizations and the associated potential cost savings in the Tuscany region. Our findings indicate that universal prophylaxis with nirsevimab, targeting all infants during their first RSV epidemic season, substantially reduces RSV-related hospitalizations (*n* = 378), with a benefit–cost ratio close to break-even (0.96). While alternative, more selective, prophylaxis strategies with nirsevimab were more cost-effective, they prevented fewer hospitalisations. This supports the value of implementing prophylaxis on a broad scale from a public health perspective.

Consistent with the recommendations of the Life Calendar Vaccination Board and the Italian Society of Neonatology (24), our analysis hypothesized nirsevimab prophylaxis programs that would cover the entire cohort of newborns during their first RSV season. The cost–benefit of implementing three different possible prophylaxis strategies with nirsevimab were evaluated. The impact of these strategies was evaluated using real-world data from an area-based cohort of infants residing in the Tuscany region during the most recent RSV season, combined with assumptions derived from published literature. The results of the study show that all possible strategies lead to significant benefits in terms of prevention of hospitalizations compared to the prophylaxis practices in place at the time of the study in the Tuscany region. A programme targeting only infants born during the season has the lowest costs, but also the lowest number of hospitalizations prevented. Considering the balance of costs and benefits, the seasonal prophylaxis programme with a targeted catch-up only for preterm infants born up to 6 months before the start of the season is the scenario with the most favorable profile. Nevertheless, the highest number of hospitalizations prevented was yielded by the seasonal prophylaxis with an extended catch-up of all infants born before the start of the season (i.e., universal strategy); even though the BCR for that strategy was less favorable, its value was very close to break-even.

Our data undoubtedly underestimate the cost–benefit of implementing prophylaxis programs with nirsevimab, especially for the extended universal strategy. Indeed, the study considered only the prevention of RSV-associated hospitalization and palivizumab discontinuation as benefits from immunization. Given to the lack of real-world data from Tuscany, the analysis did not take into account the prevention of RSV-related outcomes in the outpatient setting—such as emergency department and primary care visits—or the morbidities associated with early-life RSV infection, including the development of recurrent wheezing and asthma, as well as the healthcare economic burden arising from these chronic respiratory conditions.

Hospitalizations among infants born before the RSV season account for a substantial proportion of RSV-related admissions in our study. Consequently, the benefit derived from administering the prophylaxis in this target group is considerable: about 37% of RSV-associated hospitalizations occurred in the study population could potentially be prevented by immunizing infants born before the start of the RSV season. At the same time, the majority of RSV-associated hospitalizations occurs in infants born at term (90%). These proportions of RSV-associated hospitalizations in infants born before the season and in infants born at term align with the literature (29, 30). From the public health perspective, this highlight the importance of extended universal prophylaxis strategies designed for all infants experiencing their first RSV season, including those before the RSV season.

Cost-effectiveness studies evaluating the impact of nirsevimab prophylaxis have shown considerable variability in results, mainly related to the model structure, type of strategies compared and key input parameters, such as the price and effectiveness of nirsevimab and the RSV burden (31–36). Many studies have shown that nirsevimab prophylaxis strategies for all infants exceed commonly accepted cost-effectiveness thresholds when using list prices, and could be cost-effective if the price of nirsevimab were significantly reduced (31–36). Our sensitivity analysis confirms that the price per dose of nirsevimab is one of the most influential factor on the cost–benefit of the prophylaxis strategies. In most studies, the economically justifiable price of nirsevimab was found to be in the range of about 100€ to 300€ (31–36). Our study, based on the actual negotiated price of nirsevimab (i.e., 230 € per dose), suggests that the threshold for economic justification of universal prophylaxis strategies likely falls within this range.

In addition to the price per dose of nirsevimab, the exploration of parameter uncertainty through sensitivity analysis identified the cost of treating an RSV-associated hospitalization and the efficacy of nirsevimab as key drivers of the cost–benefit of the prophylaxis strategies analyzed, consistent with findings in the literature (33–35). It is important to note that the values of these two parameters are likely to be highly accurate, thereby reducing the level of uncertainty in the findings. Specifically, the hospitalization cost data are based on actual real-world estimates collected from the target population, while the efficacy of nirsevimab is consistently and robustly confirmed by evidence coming from the real-world settings (17, 18, 21, 37), which aligns closely with data obtained from clinical trials (14–16).

The strength of this study lies in its reliance on updated real-world data, complemented by assumptions from relevant literature. Hospitalization incidence and associated costs, as well as costs related to prophylaxis with palivizumab, were derived from the comprehensive monitoring of a population-based full birth cohort experiencing their first RSV epidemic season. Furthermore, the cost–benefit evaluation of the intervention was conducted using actual data from this cohort and the negotiated acquisition price of nirsevimab by the Tuscany Region. Moreover, the study considered infants experiencing their first RSV epidemic season during the most recent season (2023/2024), during which the RSV epidemiological situation stabilized following the disruption caused by the COVID-19 pandemic and the implementation of non-pharmacological interventions during the 2020/2021 season (38). In the Tuscany Region, surveillance efforts were significantly intensified following the resurgence of RSV during the 2021/2022 season (39–41). Consequently, the data utilized in this study provide a more accurate assessment of the actual burden of RSV-related hospitalizations in the infant population within the region.

The study has several limitations. First, this analysis focused exclusively on RSV-associated hospitalizations due to the lack of real-world data on other RSV-related outcomes in the Tuscany Region, such as emergency department visits or primary care consultations. This not only underestimated the potential benefits of prophylaxis with nirsevimab, as described earlier, but also limited the feasibility of conducting a cost-effectiveness analysis using more synthetic and comprehensive measures, such as quality-adjusted life years (QALYs), to evaluate them. Secondly, certain assumptions made in this study will need to be revisited as new evidence emerges from the literature. Specifically, the protection offered by nirsevimab was assumed to remain constant over time. However, given that antibody levels decline over time, it is plausible that the protection conferred by nirsevimab may gradually decrease following a gradient. At present, the exact decay kinetics remains unknown. Another factor concerns the indirect effects of the intervention on RSV transmission within the non-immunized population. The impact of nirsevimab on herd immunity has yet to be thoroughly explored, and future economic evaluations should incorporate such effects if relevant evidence becomes available. The benefits of immunization could be even greater if relevant herd immunity and reduced virus circulation within the community are confirmed. Thirdly, the analysis did not consider the occurrence of serious adverse events following nirsevimab administration. However, this omission is unlikely to have significantly impacted the estimates due to the rarity of such events. Lastly, the study adopted the perspective of the healthcare payer, which does not fully capture the broader benefits of a prophylaxis program. For instance, it did not account for indirect “social costs” associated with RSV-related hospital admissions, such as parents’ lost workdays, or the potential alleviation of winter pressures on the healthcare system. This includes benefits such as reducing postponed surgical procedures caused by critical bed shortages, which could represent an additional indirect advantage of prophylaxis strategies.

Universal prophylaxis strategies with nirsevimab, targeting all infants experiencing their first RSV epidemic season, significantly reduce the burden of hospitalizations within this population without increasing the economic strain on the healthcare system compared to prophylaxis practices using palivizumab, provided that a negotiated price for nirsevimab—substantially reduced compared to the list price—is considered. The implementation of such strategies appears even more justifiable when accounting for the broader benefits of immunization. These include direct advantages, such as the prevention of RSV-related outcomes in outpatient settings, the reduced risk of developing chronic conditions following RSV infection, the alleviation of pressure on healthcare systems, and the wider societal benefits.

Further studies considering the exact duration of protection and herd immunity will be necessary once evidence emerges from the literature. Moreover, once more definitive real-world data become available, direct head-to-head comparisons of the various nirsevimab strategies will be needed to determine the approach that maximizes cost-effectiveness. Additionally, prospective studies using real-world data will be required after the implementation of prophylaxis to monitor the RSV epidemiological scenario.

## Data Availability

The raw data supporting the conclusions of this article will be made available by the authors, without undue reservation.

## NETVAC Group

Rino Agostiniani, Nicoletta Ambrosino, Roberto Bernardini, Alessandra Buracci, Renata Cardamone, Angelo Cardiello, Sara Casalini, Barbara Castelli, Flavio Civitelli, Roberto Danieli, Susanna Falorni, Giovanni Gaeta, Luigi Gagliardi, Salvatore Grosso, Elisa Guidoni, Pamela Liuzzo, Marco Lucherini, Letizia Magi, Graziano Memmini, Gianpaolo Mirri, Marco Antonio Motisi, Caterina Pagano, Walter Maria Sarli, Giovanni Suriano, Angelina Vaccaro, Pier Luigi Vasarri, Marina Vignoli.
