# Supplementary material for: The impact of nirsevimab prophylaxis on RSV hospitalizations: a real-world cost-benefit analysis in Tuscany, Italy
Source: Front Public Health. 2025 Jul 3;13:1604331. doi: 10.3389/fpubh.2025.1604331 (PMC12267333; doi:10.3389/fpubh.2025.1604331)
Supplement: Supplementary file 1 [file Table_1.docx]

**Supplementary Table 1.** Characteristics of the RSV-associated hospitalizations occurred in the study population

|  | **Full cohort** | **In-season birth cohort** |
| --- | --- | --- |
| Gestational age at birth |  |  |
| *<32 weeks* | 7 (1.2) | 3 (0.8) |
| *32 - 33 weeks* | 9 (1.6) | 3 (0.8) |
| *34 - 36 weeks* | 38 (6.7) | 18 (4.9) |
| *≥ 37 weeks* | 515 (90.5) | 345 (93.5) |
| Median age at admission in days (IQR) | 76 (38 - 126) | 50 (29-76) |
| Age at admission in month |  |  |
| *0-2 months* | 223 (39.2) | 222 (59.8) |
| *3- 5 months* | 246 (66.7) | 147 (39.6) |
| *≥ 6 months* | 102 (17.9) | 2 (0.6) |
| Median length of hospital stay in days (IQR) | 5 (3 - 7) | 5 (4-7) |

IQR: Interquartile range
